# A common evolutionary origin for the ON- and OFF-edge motion detection pathways of the *Drosophila* visual system

**DOI:** 10.3389/fncir.2015.00033

**Published:** 2015-07-09

**Authors:** Kazunori Shinomiya, Shin-ya Takemura, Patricia K. Rivlin, Stephen M. Plaza, Louis K. Scheffer, Ian A. Meinertzhagen

**Affiliations:** ^1^Department of Psychology and Neuroscience, Life Sciences Centre, Dalhousie UniversityHalifax, NS, Canada; ^2^FlyEM Project Team, Howard Hughes Medical Institute, Janelia Research CampusAshburn, VA, USA; ^3^Department of Biology, Life Sciences Centre, Dalhousie UniversityHalifax, NS, Canada

**Keywords:** motion sensitivity, directional selectivity, lobula, medulla, lamina

## Abstract

Synaptic circuits for identified behaviors in the *Drosophila* brain have typically been considered from either a developmental or functional perspective without reference to how the circuits might have been inherited from ancestral forms. For example, two candidate pathways for ON- and OFF-edge motion detection in the visual system act via circuits that use respectively either T4 or T5, two cell types of the fourth neuropil, or lobula plate (LOP), that exhibit narrow-field direction-selective responses and provide input to wide-field tangential neurons. T4 or T5 both have four subtypes that terminate one each in the four strata of the LOP. Representatives are reported in a wide range of Diptera, and both cell types exhibit various similarities in: (1) the morphology of their dendritic arbors; (2) their four morphological and functional subtypes; (3) their cholinergic profile in *Drosophila*; (4) their input from the pathways of L3 cells in the first neuropil, or lamina (LA), and by one of a pair of LA cells, L1 (to the T4 pathway) and L2 (to the T5 pathway); and (5) their innervation by a single, wide-field contralateral tangential neuron from the central brain. Progenitors of both also express the gene *atonal* early in their proliferation from the inner anlage of the developing optic lobe, being alone among many other cell type progeny to do so. Yet T4 receives input in the second neuropil, or medulla (ME), and T5 in the third neuropil or lobula (LO). Here we suggest that these two cell types were originally one, that their ancestral cell population duplicated and split to innervate separate ME and LO neuropils, and that a fiber crossing—the internal chiasma—arose between the two neuropils. The split most plausibly occurred, we suggest, with the formation of the LO as a new neuropil that formed when it separated from its ancestral neuropil to leave the ME, suggesting additionally that ME input neurons to T4 and T5 may also have had a common origin.

## Introduction

### The Evolution of Synaptic Circuits

The problem of how a complex brain with highly organized pathways and centers could have arisen from simpler forms lacking clear structural compartments and with less differentiated cellular components, has generally attracted discussion chiefly in seeking a transition from very basal forms to those in which brain structure assumes some canonical stage (Arendt et al., 2008). With that transition comes a shift from diffuse to more centralized brains. The brains of invertebrate and vertebrate groups are said by some to have a common origin (e.g., Arendt and Nübler-Jung, 1999) and by others to have independent origins (e.g., Holland, 2003; Holland et al., 2013), while yet others invoke many origins. Thus, in the extreme case complex brains are claimed to have evolved many times (Moroz, 2012), and to have acquired differentiated neurons along independent paths (Moroz, 2009).

Some progress has been made in identifying how brain compartments might have arisen in ancestral groups. For this a generous spread of extant groups has generally to be analyzed, but still with no guarantee that all ancestral stages will be represented. For example, based on an earlier speculation (Meinertzhagen, 1991), the evolution of the optic neuropils in different arthropod groups has been suggested to have proceeded by a preexisting neuropil in an ancestral group splitting to yield two more specialized neuropils in the group’s descendants (Strausfeld, 2005, 2009). This process, which shares some features with gene duplication coupled with later diversification (Ohno, 1970), provides a powerful mechanism for brain evolution just as much as the latter does for the origins of new genes. On the other hand, it runs counter to the process of gene fusion which is proposed to occur when, during the course of evolution, two previously distinct genes fuse into a single open reading frame, and in the process optimize protein assembly by simplifying protein complex topologies (Marsh et al., 2013).

**Figure 1:**
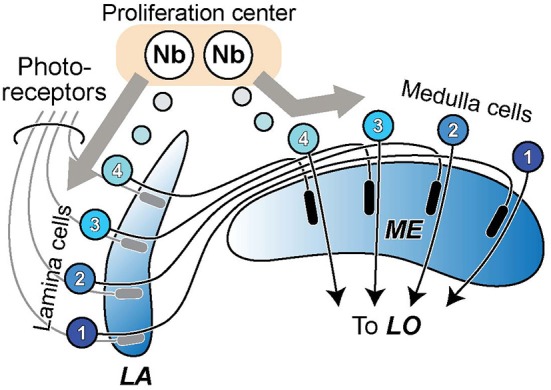
**The formation of lamina (LA) and medulla (ME) neuropils by duplication from a single proliferation center, or neuroblast (Nb) anlage.** Cells of the LA and ME cortices proliferate in antiparallel directions from this center, their axons growing in temporal sequence between the two (see: e.g., Figure 3 in Meinertzhagen, 1973). A crossed fiber tract, the external chiasma, necessarily forms between the two as the coordinated outcome of two events, cell proliferation into LA and ME cortices in antiparallel directions, and the directed growth of axons so as to penetrate the LA but grow across the face of the ME. The retinotopic sequence of innervation of the LA matches the order of projection of ME cells to the lobula (LO).

In the case of the two outermost optic neuropils in arthropods, the first optic neuropil—or lamina (LA)—and the outer part of the second neuropil, or medulla (ME), neuropil splitting is said to have occurred among neuroblast proliferation centers, or anlagen, and to be accompanied by the formation of a chiasma of axons connecting between the two new descendant neuropils (Strausfeld, 2005, 2009). The formation of the external chiasma required that LA cell axons grow down the face of the ME rather than penetrating it in a direction normal to its surface (Elofsson and Dahl, 1970; Meinertzhagen, 1973), as do photoreceptor axons that innervate the LA from the eye (Figure 1). A chiasma is in that case the inevitable topological outcome, one that preserves retinotopy but inverts it.

### The Role of Cell Duplication

Here, we address the next stage of neural evolution, namely how, once the features of their neurons had arisen in a particular animal group, circuits might have evolved within the nervous systems of animals having the same grade of brain organization.

At a yet higher level of resolution, rather less can be said about the evolution of actual synaptic circuits within any one of these neuropils, although the complexity of circuit design in the brains of extant species suggests the concatenation of many ancestral steps in this process. In an early study on a range of Diptera, changes in postsynaptic composition were identified among the fixed ensembles of neurons in each single module, or cartridge, of the LA (Shaw and Meinertzhagen, 1986). These changes can be viewed as the major avenue open to a system in which cell number is developmentally fixed, with five LA cells in the cartridges of all species examined, the five conserving both their cell identity and position. These, and other cells in the optic neuropils, have isomorphs in different species, and such examples abound. But isomorphy alone is insufficient evidence to claim that a cell type in one species is a true evolutionary ortholog of an isomorph in another. Constancy of cell number addresses this uncertainty at least in the LA, because given that the number of cells is fixed at five, the gain of a completely new cell type would otherwise have had to entail the simultaneous loss of another, previously existing type (Shaw and Meinertzhagen, 1986; Meinertzhagen and Shaw, 1989). This logic implies that the two cell types must have arisen from a look-alike cell in an ancestral LA. Many cells have similar partners, and cell duplication was probably a common route to functional divergence, along with remodeling of old circuits belonging to extinct behaviors (Arbas et al., 1991). It may therefore be significant that the paired neurites of many cartridge elements reveal a duplex arrangement that has been interpreted to reflect their duplication and deep homology. Thus in the fly cartridge the axons of LA cells L1 and L2, and two centrifugal cells C2 and C3, provide clear morphological examples of candidate duplicates, which in the case of L1 and L2 correlate with the pairing of their postsynaptic involvement at photoreceptor tetrad synapses. The ME may incorporate a similar situation but this is far less clear, with many different cell types, at least half those in the optic lobe as a whole (Fischbach and Dittrich, 1989).

At the LA’s tetrad synapses, two of the four postsynaptic sites at each tetrad are reserved for an L1 and L2 dendrite, L1 and L2 partnering each other scrupulously, using self-exclusion mediated by the redundant action of two cell adhesion genes *Dscam1* and *Dscam2* to ensure that each tetrad receives only a single dendritic contact from each cell, and that overall photoreceptor input to both is thereby closely matched at all tetrad synapses (Millard et al., 2010). The pairing of cells in the LA cartridge may be referred to as the duplication of an ancestral L-cell interneuron of photoreceptors R1-R6, to generate two sibling cell types, L1 and L2. It is important to remember however that this was not duplication by cell division, rather a change in recruitment of L-cells by the photoreceptor axon bundle (Meinertzhagen and Hanson, 1993), in a process mediated by Hedgehog (Huang and Kunes, 1996).

## Hypothesis

### T4 and T5 are Sibling Cells in Two Neuropils

All these examples consider only a single neuropil, and so far we believe that no cell type has yet been identified in two neuropils that might have arisen by the duplication of its common ancestor. Two cell types, the T4 and T5 cells of the fly’s optic lobe, provide a possible exception to this generalization, and an opening into the question of the evolutionary origins of these two interesting cells and their circuits.

To expose the many resemblances between T4 and T5 in a systematic fashion, we will first summarize their morphological similarities and highlight their chief difference, following an anatomical sequence from soma, then axon and axon terminal, then dendrites, and afterwards list their functional and circuit similarities. Finally, we give brief consideration to the presence of T4 and T5 isomorphs in flies other than *Drosophila* and the little that is known about the development of these cells. We will conclude with a brief summary of the ME input neurons to T4 and T5.

#### Morphological Similarities

The somata of T4 and T5 intermingle in the cortex of the LOP, a proposed ancestral optic lobe neuropil containing circuits for motion detection (Strausfeld, 2005). Both T4 and T5 have four subtypes (Fischbach and Dittrich, 1989) and overall in *Drosophila* there are sufficient numbers to allocate up to four representatives of T4 and four of T5 per column (Mauss et al., 2014), one of each subtype. From a soma in the LOP each T4 and T5 cell extends an axon that penetrates the LOP neuropil, and then bifurcates in the internal chiasma, with one branch that reflects and returns to the LOP to form its branched terminal. The terminal of each cell type innervates one of four strata, Lop1 (abutting the chiasma) to Lop4 (abutting the LOP cortex), as given by Fischbach and Dittrich (1989). The four subtypes are defined by the particular LOP stratum innervated by the cell’s terminal: T4a/T5a in Lop1 and T4d/T5d in Lop4. There each terminal innervates dendrites of large lobula plate tangential cells (LPTCs; Hengstenberg et al., 1982; Hausen, 1984; Douglass and Strausfeld, 2003) that signal wide-field motion in either a horizontal (HS cells) or vertical (VS cells) direction (Borst et al., 2010). The LPTCs also arborize in a stratum-specific manner in the LOP. Although it is proposed that T4 and T5 provide synaptic input to the LPTCs, for the moment this has been reported anatomically only for a single T4 input to an HS cell (Strausfeld and Lee, 1991; review: Douglass and Strausfeld, 2003).

The axon tracts between ME and lobula (LO) form a chiasma, those from neighboring transmedulla (Tm) cells inverting their retinotopic order within alternating fiber sheets, first depicted by Braitenberg (1970) as sheets 2 and 4 in his Figure 9. The fibers of T4 and T5 cells connect the neuropils between these sheets. T4 corresponds to sheet 3, and its fibers connect the ME and LOP, while T5 corresponds to sheet 1 with fibers that connect the LO and LOP. LO and LOP face each other, and unlike Tm cells, the axonal projections of the T cells do not form a chiasmal crossing between the neuropils, so that the retinal field is not topologically inverted.

**Figure 2:**
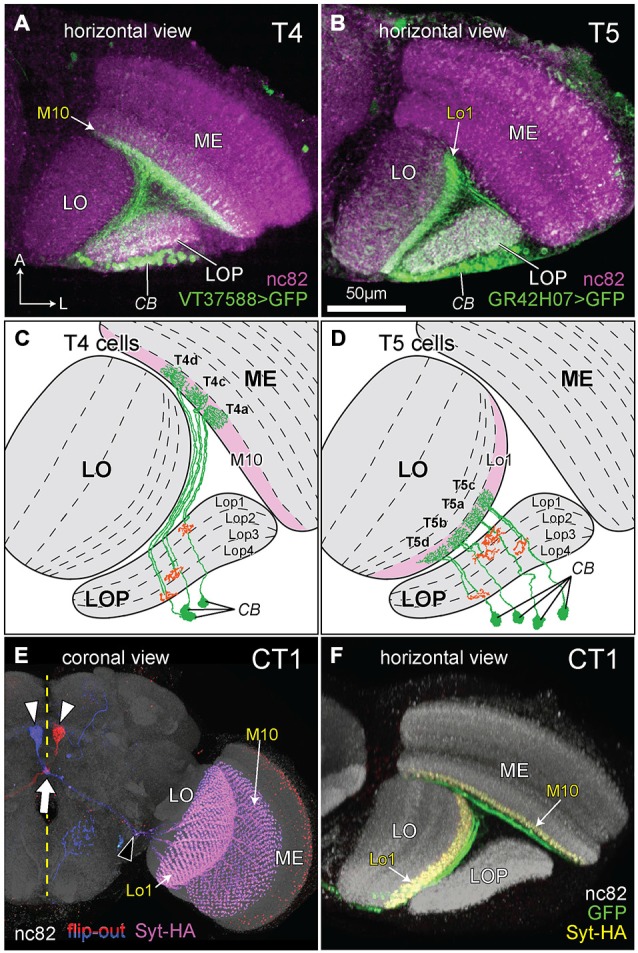
**The morphology of T4, T5, and complex tangential cell (CT1) cells. (A,B)** Innervation patterns of T4 and T5 cells. VT37588-Gal4 driven Green Fluorescent Protein GFP(T2), mCD8::GFP (LL6) highlights the dendritic zone in ME stratum M10 for T4 dendrites and GR42H07-Gal4 driven GFP(T2), mCD8::GFP (LL6) highlights the dendritic zone in LO stratum Lo1 for T5 dendrites. Cell bodies (CB) intermingle in the cortex of the LOP; ME: medulla; LO: lobula. Background immunolabel: nc82 (anti-BRP, magenta). **(C,D)** Innervation patterns of single T4 and T5 cells (after Fischbach and Dittrich, 1989). The presynaptic (output) sites of the cells in the LOP are indicated in orange. Strata housing the dendritic (input) arbors are highlighted in pink [M10 in **(C)** and Lo1 in **(D)**]. In each cell type, four subtypes (a, b, c and d) each target one of the LOP’s four strata, Lop1, Lop2, Lop3 and Lop4. Only three T4 subtypes are shown in **(C)**, as originally illustrated by Fischbach and Dittrich (1989). **(E)** Coronal projection of the CT1 cell. A bilateral pair of CB lies one each on either side of the midline (yellow dashed line), somewhat dorsal to the antennal lobes (white arrowheads). The axons project contralaterally, crossing each other at the midline (arrow) and medial to the optic lobe they bifurcate (black arrowhead) into a ME and a LO fiber. Columnar terminals are visible as a repeated array in strata M10 and Lo1. Blue and red: single cell flip-out clone for each CT1 cell (Nern et al., 2015); magenta: synaptotagmin-hemaglutinin (Syt-HA) indicating presynaptic sites; background immunolabel: nc82 (gray). **(F)** Innervation of CT1 in the optic lobe, seen in a horizontal plane. A single CT1 cell innervates the M10 and Lo1 strata, both in their entirety, communicating with T4 and T5 cells, respectively. Its axons run over the surface of the neuropils and project into them. A presynaptic marker Syt-HA expresses throughout both strata. Green: CT1-Gal4 driven GFP; yellow: Syt-HA; background immunolabel: nc82 (gray).

The morphologies of T4 and T5 cells are highly characteristic, and each has unmistakable similarities to the other (Figures 2A–D) especially in the trajectory of its axon and the branching pattern of its dendrites. In fact, morphological similarities between the branching patterns of their dendritic arbors only make it more clear of the major difference between T4 and T5: the location of the arbor itself. This invades ME stratum M10 in the case of T4 or LO stratum Lo1 for T5 (Figures 2C,D).

In a further final similarity, T4 and T5 cells are both also innervated by a complex tangential cell (CT1), a newly discovered giant contralateral tangential neuron from the central brain. This cell has laterally directed neurites that split and innervate terminals in both the ME and LO, one per column in all columns of the M10 and Lo1 strata (Nern et al., unpublished), and that receive reciprocal input from the corresponding T4 (ME) and T5 (LO) cells (Figures 2E,F; Takemura et al., unpublished).

#### Functional Similarities

T4 and T5 are also functionally related. Both are part of the fly’s motion sensing apparatus, and recent studies in *Drosophila* obtained using semi-automated serial electron microscopy (EM) reconstruction of synaptic circuits (Takemura et al., 2011, 2013; Shinomiya et al., 2014), in conjunction with two-photon calcium imaging (Akerboom et al., 2012) and genetic dissection (Rister et al., 2007; Gao et al., 2008; Simpson, 2009) strategies, have led to the functional analysis of networks that is uniquely possible in this species (Meinertzhagen and Lee, 2012). Applied to T4 and T5, these approaches reveal that both cell types exhibit direction-selective responses (Maisak et al., 2013). Both are directionally tuned to one of four cardinal directions of motion: upward and downward, front-to-back, and back-to-front, the preferred direction depending on the particular stratum of the LOP in which each subtype terminates.

Blocking activity in both T4 and T5 in *Drosophila* abolishes LPTC motion responses (Schnell et al., 2012). T4 and T5 do however segregate functionally with respect to contrast polarity: whereas T4 cells selectively respond to moving brightness increments (ON-edges), T5 cells only respond to moving brightness decrements (OFF edges; Maisak et al., 2013; Borst, 2014). Thus, when the output from either T4 or T5 cells is blocked, the responses of postsynaptic LOP neurons to moving edges are compromised, but for ON- (T4 block) or OFF-edges (T5 block; Maisak et al., 2013).

Both cell types express a cholinergic phenotype, at least in *Drosophila*. They exhibit immunoreactivity to choline acetyltransferase (ChAT), but a lack of immunoreactivity to the vesicular GABA transporter VGAT (Mauss et al., 2014), and they express ChAT transcripts, thus suggesting a cholinergic phenotype at least in *Drosophila*, but with different acetylcholine receptor transcripts that presumably reflect differences between the cholinergic inputs they receive (Shinomiya et al., 2014). In addition, using pharmacological and optogenetic approaches, Mauss et al. (2014) have shown that LPTCs are very likely to receive direct cholinergic inputs from T4 and T5.

#### Developmental Similarity

A further recent piece of evidence links T4 and T5. During development, progenitors of both cell types express the proneural gene *atonal* early in their proliferation from the inner anlage of the developing optic lobe, and these two cell types are alone among many other progeny to do so (Oliva et al., 2014). Remarkably, *atonal* in these cells does not act as a proneural gene serving to convert the fate of an ectodermal cell to a neural derivative, as would be the case if they were to resemble photoreceptors and chordotonal organs of the peripheral nervous system (Jarman et al., 1993, 1994). Rather, it is required specifically in the inner anlage among particular neural progenitors to regulate neurite outgrowth exclusively in their neuronal progeny, T4 and T5. That outgrowth contributes to the formation of the fiber bundles between the neuropils, T4 fibers between the ME and LOP, and T5 between the LO and LOP. The expression of *atonal* suggests that transcriptional programs initiated specifically in the T4 and T5 progenitors are necessary for subsequent neuronal morphogenesis in these two cell types, and that those programs are similar in the two types, just as are the trajectories of their neurites and the pathways these both establish in the internal chiasma.

Representatives of T4 and T5 have been reported in a wide range of Diptera (Buschbeck and Strausfeld, 1996), and bushy T-cell counterparts exist in many insect groups, recognized a century ago in honeybees by Cajal and Sánchez (1915), and even in malacostracan crustaceans which diverged from the insect line about 425 million years ago (Strausfeld, 2012), so that T4 and T5 cell orthologs almost certainly predated the origin even of insects.

#### The Medulla Input Neurons to T4 and T5

**Figure 3:**
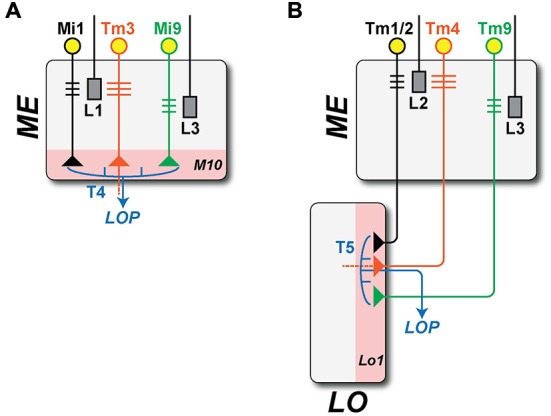
**Brightness increment (L1: ON-edge) and decrement (L2: OFF-edge) detecting pathways.** Information on moving ON- and OFF-edges is mediated by anatomically distinct pathways that originate with the ME terminals of two LA cell types, L1 and L2. In the ME, both provide input to relay neurons that are either unicolumnar (L1: Mi1; L2: Tm1/2: black) or multi-columnar (L1: Tm3; L2: Tm4: orange, note wider dendrites). Both provide input in turn to neurons T4 and T5 (blue), that integrate information from both the uni- and multicolumnar ME relay neurons and that project in turn to the LOP. T4 and T5 have postsynaptic dendrites in single layers of their respective neuropil (pink): strata M10 in the ME and Lo1 in the LO. Both also receive minority inputs from additional pathways that originate with a third LA cell type L3 (T4: Mi9; T5: Tm9; green). **(A)** ON-edge (L1 + L3 to T4) pathway. The proximal axon of Tm3, which projects from the ME to Lo4 of the LO, is omitted (dashed line). **(B)** OFF-edge (L2 + L3 to T5) pathway. The proximal axon of Tm4, which projects to deeper strata of the LO, is likewise omitted (dashed line).

Given the locations of their respective dendritic arbors, T4 and T5 receive inputs from different ME cells (Figure 3). T4 has major input from two of L1’s ME target neurons, ME intrinsic neurons Mi1, which are unicolumnar (receiving L1 input from a single column only), and transmedulla neurons Tm3, which are multicolumnar (receiving L1 input from several neighboring columns; Takemura et al., 2013). T5 has its major inputs from the following ME targets of L2: unicolumnar transmedulla neurons Tm1 and Tm2 (Takemura et al., 2011; Shinomiya et al., 2014), and multicolumnar transmedulla neurons Tm4. Tm1 and Tm2 are often postsynaptic co-occupants at the same synapses from L2 cells in ME stratum M2 (Takemura, unpublished).

Each pathway thus receives input from ME neurons that are both unicolumnar: Mi1 for T4 and Tm1/Tm2 for T5; and multicolumnar: Tm3 for T4 and Tm4 for T5 (Figure 3). In a final similarity, both T4 and T5 also receive input from an L3 pathway via unicolumnar ME neurons (Figure 3): Mi9 for T4 (Takemura et al., 2013, unpublished) and Tm9 for T5 (Takemura et al., 2013; Shinomiya et al., 2014).

### Some Consequences: if T4 and T5 were Evolutionary Sibling Cell Types

**Figure 4:**
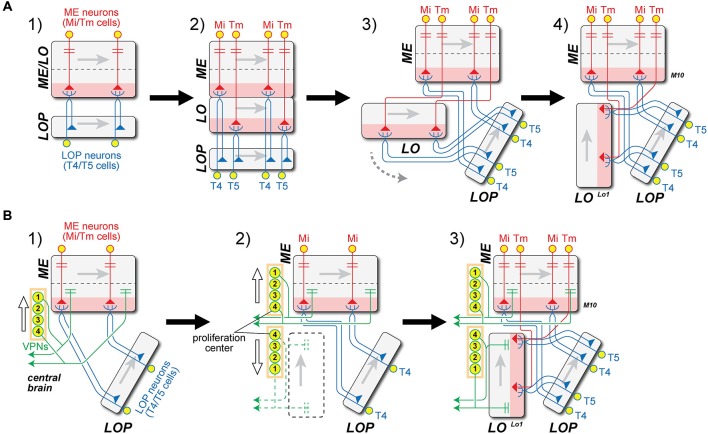
**Two models for the origin of the lobula (LO) and internal chiasma. (A)** (1) Hypothetical ancestral form. Medulla (ME) neuron antecedents to Mi and Tm neurons (red) in the ancestral ME/LO receive input in distal strata and provide input to undifferentiated ancestral T4/T5 LOP cells (blue). These cell pairs constitute two combined classes that are not initially differentiated, but which later duplicate. Gray arrows in each neuropil indicate direction of accretion of new columns to the neuropil, from first to last, corresponding to the posterior-anterior axis of the visual field; (2) T4/T5 cells duplicate, and their dendritic zone (pink) segregates into two. Then ingrowing Tm terminals separate the two layers between what will become the M10 and Lo1 strata, one for each differentiated T4 and T5 cell type, respectively. The original ME and the newly developed LO strata are thus arranged in tandem; (3) The newly formed LO separates from the ME, segregating T5 from T4 cell populations, and generating what will become the internal chiasma; (4) The LO rotates 90° in a counter-clockwise direction (gray dashed arrow in A3), the Tm axons (red) generating a chiasma between the ME and LO. Now the Tm cell axons must have changed the direction of their entry to the LO, to enable the LO to become rotated to its current position, parallel to the LOP. This causes the chiasma to form between ME and LO. **(B)** Alternative “VPN-duplication” model. Model A offers an explanation for the close similarity between ME stratum M10 (with T4 dendrites) and LO stratum Lo1 (with T5 dendrites), but not for the chiasma of Tm cell axons between them. (1) Ancestral form as in A1, but with additional projections from ancestral columnar VPNs in the proximal ME to the central brain. The numbers on the VPN CB represent the order of cell generation from a proposed proliferation center (1 = early, 4 = late). The direction of cell displacement is shown by a white arrow; (2) Some classes of VPNs may have duplicated in an anti-parallel direction (white arrows), originating from the proliferation center. It is proposed that the formation of a new neuropil, the LO (dashed black box), was induced by newly generated VPN populations. The polarity of the LO could then be defined by the order of VPN generation, as in the ME (Figure 1); (3) LO is now innervated by Tm cells from ME, which were generated by the duplication of Mi cells, as well as by T5 cells from LOP. Insofar as the anterior-posterior axis is inverted in LO from ME, the axons of Tm cells have to cross to innervate LO, causing the inner chiasma to form.

From the weight of evidence presented above, we next propose that T4 and T5 are in fact evolutionary siblings that derived from a common ancestral cell population, and that it is this path of descent from a single ancestral T4/T5 cell type that supports their deeper similarities, rather than, say, the functional roles that each cell type had to play to generate opponent ON- and OFF-edge motion pathways. A number of issues immediately present themselves: (1) how the LO arose, and whether, as we are about to suggest, this could have been from an ancestral neuropil fused with what then became the modern proximal medulla (PM); (2) the topological requirements for this transition, especially those of axon trajectories within the internal chiasma (Figure 4); and (3) the evolution or co-option of T4 and T5’s input neurons especially from different types of ME cells. We now consider these three questions in greater detail.

#### The Origin of the Lobula From an Ancestral Neuropil Fused with the Medulla

First, the population of a single ancestral cell type T4/T5, which had dendrites in an antecedent Pm at a level corresponding to the modern stratum M10 could have been duplicated, when the ancestral neuropil widened in a proximal direction, to yield a separate, independent ME and LO (Figure 4A). The two classes of cells would have had dendrites in parallel with each other (Figure 4 panel A2). The newly formed LO would then delaminate from the ME to form two separate new cell populations, T4 for ME and T5 for LO. It is not clear, however, whether the T4/T5 segregation might have preceded neuropil splitting, possibly when “ON”- and “OFF”-edge pathways segregated, or* vice versa*.

Independent of the LO’s origin by delamination from the Pm that we propose here, the LO is also a derived neuropil in another important way. Its motion sensing partner neuropil in flies—the LOP, is considered ancestral (Strausfeld, 2005), but in early insect groups such as dragonflies, and persisting in hymenopterans (Strausfeld, 2012), the LOP proper is not clearly separated from the LO (Strausfeld, 2005). Instead of a sublobula some insects have a satellite LO instead of a LOP (Strausfeld, 2005). These alternative neuropil arrangements involving the LO do not bear on the segregation of sibling T4 and T5 cells proposed here and could not easily have generated sibling T4 and T5 cells during a single duplicative step, but may nevertheless have predated the delamination of the LO from the Pm. The latter possibility is consistent with the notion that the ME is actually a compound neuropil, with an outer ME receiving input from LA cell terminals, and an inner ME developmentally related to the LO and LOP. Supporting this relationship is the fact that: (a) the arrangements between the inner optic neuropils in different insect groups can suggest their developmental continuity. Thus in Ephemeroptera, the LO is partly fused to the LOP, while in Trichoptera the inner ME is connected during development with the LOP (Strausfeld, 2005); and in *Drosophila*; (b) the eyeless *sine oculis* mutant lacks both LA and outer ME, whereas the inner ME, LO and LOP are all still present (Fischbach, 1983); and the tips of the outer anlage of the developing optic lobe produce neurons that populate all three neuropils, ME, LO and LOP (Bertet et al., 2014).

Apart from the similarity of their inputs to motion pathways in flies, the two modern structures, the Pm part (strata M8–M10) and the new LO, share some significant anatomical features in common. These include a population of visual projection neurons (VPNs) conveying visual inputs to the central brain, and the nearly-symmetrical innervation of both neuropils by LOP Y cells (Fischbach and Dittrich, 1989). The Pm is separated from the distal ME (M1–M6) by the serpentine layer (M7), a fiber-rich stratum which houses a significant number of tangential axons. Some types of ME neurons, such as Pm cells, T3 cells and Y cells, only project to the Pm and never penetrate its distal strata, while input neurons such as the R7/R8 photoreceptors and LA cells, and the distal ME (Dm) cells, only innervate the distal ME. These anatomical features imply that the LO is much more strongly related in its cellular composition to the Pm than to the distal ME, a difference that can be interpreted in the context of both the ME’s development and evolution. Thus in our interpretation the LO may have arisen, and so can be considered, as a duplicated structure of the Pm, to which it may initially have been fused in ancestral forms.

We find these “duplication models” the easiest way to account for how two such similar cell types as T4 and T5 could be located in modern forms in these two neighboring neuropils. Even so, we acknowledge that no extant arthropod group has yet been reported that represents an intermediate condition with an ancestral fused neuropil, prior to its separation into ME and LO. Although we find this omission inconvenient, we do not think it damaging to the theory, because of the relative lack of diversity in optic lobe organization among extant groups anyway (Strausfeld, 2005), and because of the paucity of the fossil record and the understandable difficulties in interpreting neuroanatomy from examples it provides. Recent studies do now begin to address this omission (e.g., Ma et al., 2012) however, although three nested optic centers have not been explicitly homologized to LA, ME and LO neuropils in the brain of *Fuxianhuia*, the earliest Cambrian arthropod ever examined. We also note that the process of neuropil splitting we propose may differ from the duplication of cell lineages in the inner proliferation zone previously proposed to have given rise to the LO as a novel neuropil (Strausfeld, 2005).

The process of neuropil splitting we propose to explain similarities between T4 and T5 as evolutionary siblings may be compared with a scheme previously suggested to have given rise to the LO as a novel neuropil (Strausfeld, 2005). In that case the early duplication of a cell lineage that originally produced the ancestral LA is proposed to have given rise to an outer and inner neuropil, the modern LA and ME, in a manner long known and depicted here in Figure 1, so as to give rise to the external chiasma. The resulting ME is proposed still to have retained its uncrossed projection to the LOP (Strausfeld, 2005). A duplication of cell lineages in the inner anlage of the developing optic lobe is next proposed to have given rise to the LO as a novel neuropil (Strausfeld, 2005), in a step we propose instead to have arisen by its cleavage from the Pm. The internal chiasma is then proposed by Strausfeld (2005) to have derived from uncrossed axons originally supplying the LOP that subsequently supply collaterals to the opposing surface of the newly evolved LO. Strausfeld’s (2005) view of the LO’s origin is that it arose as a duplication of cell lineages in the inner optic anlage, whereas our view of the LO’s origin, as the product of a neuropil cleaved from the Pm, is based solely on evidence from similarities between T4 and T5. It may therefore be instructive to seek orthologs for T4 and T5 in the optic lobe of the ancestral apterygote *Lepisma*, which is thought to lack a LO (Strausfeld, 2005).

#### The Axon Trajectories within the Internal Chiasma

The axons of both sibling T4/T5 cell types would have had to negotiate the internal chiasma, a newly formed fiber tract between ME and LO, to which they contribute. As a result of the split, the original T4/T5 population with a single stratum of dendrites would then have become segregated, their now separate dendrites contributing to two new neuropil strata that would become a separate ME stratum M10 and LO stratum Lo1. The dendrites of the two new cell types extend into the depths of their respective neuropils, and thus in opposite, antiparallel directions to each other (Figure 4 panel A4). The T4 population alone could retain this ancestral projection to the LOP, while the T5 pathway would have been novel. Although this change would have endowed species that predated the fly with additional processing strata, it is not in fact clear what might have impelled a split in the ancestral fused neuropil, and with it the retina (RE)-routing of Tm cell axons (such as those from input neurons to T5) to form a chiasma. On the other hand it is easy to imagine how by the growth of their axons the inputs to both newly segregated cell types, as separate T4 and T5 cells, could have become specialized and how by extending their axons to new terminal locations Mi cells could form the many different classes of new Tm cells, by extending axons to the LO when that neuropil drew apart from the ME.

We propose a scenario, one of several that could have generated the requisite duplication (Figure 4A), with a chiasmal fiber tract arising after the new LO was repositioned by a rotation in a counter-clockwise direction (Figure 4 panel A3). This would have required that the Tm cell axons change their direction of growth to penetrate the new LO in a direction from its distal to proximal strata (Figure 4 panel A4). Thus the process of fiber growth could therefore not have arisen in a similar manner to that described above for the external chiasma (Figure 1).

Given the problems raised by fiber growth, we therefore consider as an alternative that a quite different transition for the new LO could have been driven by Cell bodies (CB) in the central brain (Figure 4B). We first compare the correlation between the retinotopic organization of optic neuropils and the sequence of cell proliferation generating their columnar elements that was first described for the external chiasma (Figure 1). The ME and LOP both have their own populations of neurons that mainly project to their respective neuropils; Mi/Tm/TmY and Dm cells in the ME, and T4/T5 cells in the LOP. This organization corresponds to that of the LA and ME, but denying any closer similarity differs insofar as the axon tracts between them are uncrossed. In the case of the LO, however, only a few cell types that arborize mainly in this neuropil and not elsewhere, have actually been reported. These include LO intrinsic (Li) cells (Fischbach and Dittrich, 1989; Lin et al., in press).

The only significant cell population with a major innervation site in the LO is in fact that of the VPNs, which are actually not optic lobe neurons at all, but develop from neuroblasts in the central brain (Ito et al., 2013; Yu et al., 2013; Awasaki et al., 2014). The LO houses a large number of terminals from multiple classes of these VPNs (Otsuna and Ito, 2006). Their mode of proliferation has yet to be reported, but we suggest that some classes of VPNs may have duplicated in an anti-parallel direction (arrows, Figure 4 panel B2), originating from a proliferation center in the lateral cell body rind. We propose the existence of such a center because many VPNs, the LO columnar (LC) cells, provide retinotopic columnar inputs to the LO and send their outputs to central brain neuropils. By analogy with the columnar neurons of the LA and ME we postulate that retinotopic input was required to generate the retinotopic order of LC cells. Although no direct evidence has so far shown the mode of their production, we propose that these neurons developed from a proliferation center. Axon projections from the duplicated VPNs contributed, we suggest, to the formation of the new synaptic neuropil, the LO, and axons from both the ME and LOP then projected into the LO and innervated its columns. Tm cells and T5 cells then differentiated from preexisting ME and LOP neurons, respectively. The polarity of the VPNs is inverted in the LO with respect to the ME, so that the projection of axons between the two neuropils necessarily formed a chiasma (Figure 4 panel B3). This model enables the topologically demanding requirement for a chiasma to form between ME and LO but not between either of the other neuropil pairs: neither ME and LOP, nor LO and LOP.

While many identified VPNs innervate the LO, the ME also houses terminals of a number of VPNs, including both columnar and tangential neurons (Fischbach and Dittrich, 1989; Raghu and Borst, 2011; Otsuna et al., 2014). In the ME and LO, however, known VPNs communicate neither with T4 in M10 nor with T5 in Lo1. These similarities suggest that some ME and LO VPNs are homologous, and share the same evolutionary origins, which if it were true would imply that VPNs already existed at an evolutionary stage corresponding to that shown in Figure 4 panel A2, and also duplicated when the upstream Mi/Tm cell population duplicated.

As a yet further possibility, a single ancestral population of T4/T5 cells may have had dendrites in an ancestral neuropil that eventually duplicated to produce the ME and LO, but the LO remained fused to the LOP until T4 and T5 neurons had become segregated from each other. This configuration could have corresponded to an intermediate stage in which, as Strausfeld (2005) points out for species of Ephemeroptera, projections from the inner ME formed a chiasma with a LO that was contiguous with a sheet of neuropil supplied by uncrossed axons from the ME, an arrangement that suggests an intermediate stage in the relocation of the ancestral LOP from its original position opposite the LO to one beneath it.

### Evolution of the Input Pathways to T4 and T5

We now explore how the inputs to the new sibling T4 and T5 cell types might also have changed when these two cell types segregated from each other. We first assume that the original T4/T5 cell neurons were an ancestral substrate for motion detection, given that the T4 and T5 descendants both retain this function, but that at some stage T4 and T5 became specialized to detect ON-edges and OFF-edges, respectively (Joesch et al., 2010; Eichner et al., 2011; Maisak et al., 2013). During the transition, to retain their essential function in motion detection, it seems more likely that the inputs to T4 and T5 inherited from ancestral arthropod species would have evolved from existing inputs, rather than that completely new inputs arose. However the exact sequence would depend on which of the modes proposed above was used to generate separate T4 and T5 classes, and whether the specialization of T4 and T5 predated the split in their neuropil or followed after it.

**Figure 5:**
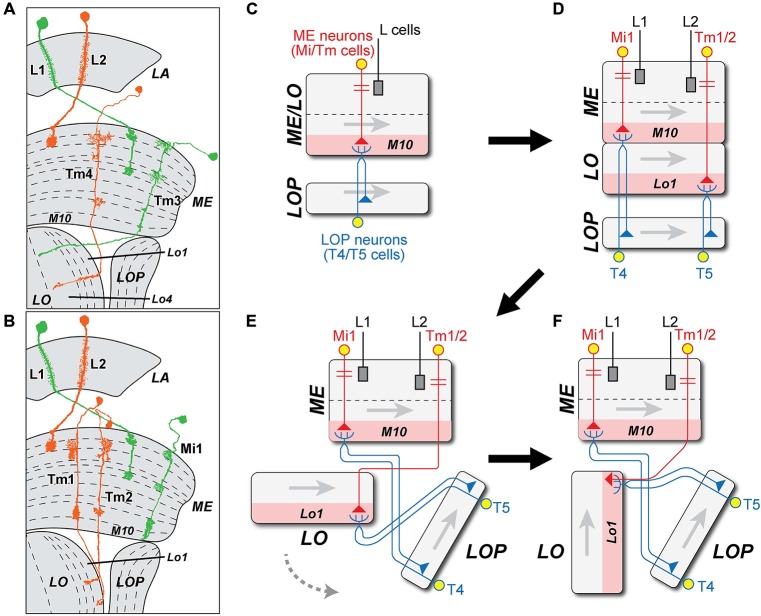
**Possible origins of ME cell inputs to T4 and T5. (A,B)** Profiles of Mi1 and Tm3 (L1 pathway, green) and Tm1, 2 and 4 (L2 pathway, orange), from Golgi impregnations (after Fischbach and Dittrich, 1989). Tm3 and Tm4 are proposed to have arisen by duplication, and extend axons to terminals in the LO. **(C)** The original T4/T5 cell population of an ancestral fused ME/LO is proposed to have received input from an ancestral ME intrinsic (Mi) neuron from which later Tm cells derived. Gray arrows indicate direction of accretion of new columns to the neuropil, corresponding to the posterior-anterior axis of the visual field (cf. Figure 4A). **(D)** When the LO separated from the inner layers of the ME, Tm1 and Tm2 cells are proposed to have derived from the Mi cells, losing their connection with the T cells in stratum M10 but retaining synaptic connection with newly differentiated T5 cells in LO stratum Lo1. The Mi cell type survived as Mi1. **(E)** The LO split from the ancestral ME. The axon of the Tm1/Tm2 cells entered the LO from its distal end and terminated in Lo1, located on one side of the ME. **(F)** Tm cell axons enter the LO from the side of the LOP. In a final step, Tm1/Tm2 segregate into two separate Tm cell types, both retaining input from L2.

The easiest case to imagine is that in the model shown in Figure 4A, because the dendrites of both new cell types would initially have been closely associated, and at least some input neurons to T4 and T5 should be equivalent sibling descendants. The Tm3 inputs to T4 and the Tm4 inputs to T5 provide the most obvious case, with Tm3 providing input in stratum M10 and Tm4 in stratum Lo1 (Figure 5A). Both also provide inputs to the deeper LO stratum Lo4, but to unknown targets there (for Tm4: Shinomiya et al., 2014). We suggest that these two cell types arose by duplication from a single input innervating the ancestral T4/T5 population in the antecedent ME, each cell type then developing its own individual dendrites and arbors in the ME and deeper in the LO, and eventually terminating in stratum Lo4 (Figure 5A). Mi9 and Tm9 input is another obvious case (Figure 3). Suggesting their common ancestry, both ME cells receive input from the same LA cell type, L3 (Takemura et al., 2013).

Tm3 provides input to T4 that is proposed as one arm of an elementary motion detector (EMD) circuit, comparing this against an input from a unicolumnar partner, Mi1 (Takemura et al., 2013). The role of Tm4 input to T5 is yet to be resolved (Shinomiya et al., 2014). At least for Tm3, however, a second input to T4 must have survived the proposed split between ME and LO, in order to retain a functional EMD circuit.

What second input would the ancestral T4/T5 cell population have had? Once the T4 and T5 cell populations had separated, their unicolumnar inputs (which became Mi1 for T4, and Tm1/Tm2 for T5) diverged further from each other than Tm4 did from Tm3. Two broad options for a unicolumnar input exist: (1) Either there was a single input terminal in each column, one that terminated in ME strata M9, M10, and possibly Lo1, and this became Mi1 for T4, with its own input in turn from the L1 pathway. For input to a new T5 cell population from the L2 pathway, an Mi1-like ancestor would then have had either to extend deeper to LO stratum Lo1 or to retract selectively from M9 and M10 (Figures 5B–F), and this would also require it to have negotiated the chiasma between LO and ME. For this, we find most plausible an intermediate stage in which the T4 and T5 cell populations first specialized and then split, before the ME and LO neuropils separated, and a chiasma grew between the two (Figure 4). Finally, Tm9 on the L3 pathway grew to the LO, mimicking the Tm1/Tm2 inputs; (2) In an alternative sequence, a single input neuron that innervated the combined M10/Lo1 stratum duplicated to yield sibling inputs. These then survived as Tm1 and Tm2 innervating T5 cells in Lo1, so that the two preserved many similarities, especially at their input synapses from L2 (Takemura et al., 2011, 2013). In M10, one was then lost and the other retracted from Lo1, leaving the surviving cell type Mi1 innervating T4.

The options for the “VPN-duplication” model (Figure 4B) are much wider and await evidence on the pattern of their cell production.

**Figure 6:**
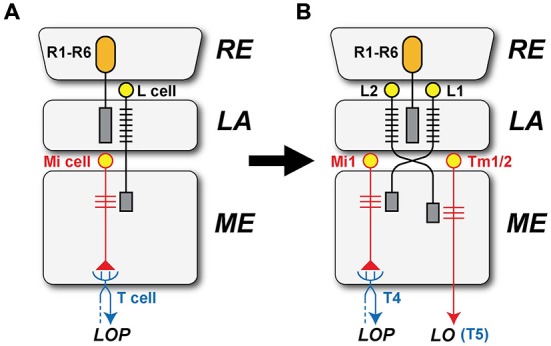
**The separate origins of the L1 and L2 pathways. (A)** L1 and L2 are proposed to have originated by a duplication event involving their innervation by photoreceptor axons derived from the retina (RE). In the ancestral form, only a single subtype of LA (L) cell is proposed to have received input from the photoreceptor cells (R1–R6) in the LA and provide output to the ME, later through the external chiasma. Ancestral unicolumnar Mi cells are shown as a target of the L-cell terminal in the ME, and these provide input to a single type of ancestral T-cell that transferred motion information from the ME to the LOP, in parallel with the segregation of separate T4 and T5 cells. **(B)** L1 retained an ancestral unicolumnar target neuron that became Mi1, together with multicolumnar neurons that transformed into Tm3 neurons after the ancestral combined neuropil split to yield separate ME and LO neuropils. The ancestral T-cell differentiated into T4 cells, which mediate signals in the L1 pathway. L2’s target neurons transformed into Tm cells, one undergoing a further duplication to yield paired unicolumnar neurons Tm1 and Tm2, and Tm4, which transformed from an ancestral multicolumnar Mi neuron. The Tm cells extended axons to the newly generated LO, providing input to the T5 cell.

Did L1 and L2 arise by duplication in the LA to initiate the segregation between T4 and T5? The circuits driving modern T4 and T5 differ in receiving respective inputs from the L1 and L2 pathways. How might these have arisen as separate pathways? Evidence is mostly only suggestive, but both L-cells are similar in two important ways. First, their dendrites cooperate closely in occupying two of the four postsynaptic sites at all photoreceptor tetrads (Meinertzhagen and O’Neil, 1991; Rivera-Alba et al., 2011), and second their axons are similar in occupying paired locations at the cartridge axis, in positions determined by the matched levels of their N-Cadherin (nCad) expression (Schwabe et al., 2014). Meinertzhagen and Shaw (1989) have previously proposed that the paired nature of L1 and L2 may reflect an original duplication event in these pathways (Figure 6), possibly so as to match the transcriptomes of these cells more closely to each other than to other LA cells, but certainly any duplication must have been long ago, presumably predating the segregation of T4 and T5 populations. Could a duplication in the L1 and L2 pathways have driven the downstream segregation between the T4 and T5 cells? Neurons homologous to L1 and L2 date back long periods of time even by geological standards. For example tetrad synapses and L1 and L2 counterparts also exist in the optic lobe of locusts (Wernitznig et al., 2015). So these features are now suggested to have developed in a common ancestor and be widely shared across neopteran species, probably dating back at least ~390 million years in the Devonian (Misof et al., 2014). Their proposed duplication has also been accompanied by a change in neurotransmitter phenotype, glutamate for L1 and acetylcholine for L2 (Takemura et al., 2011).

### The Voice of Neurotransmitters

Finally, what can be learned from the neurotransmitter phenotype of these cells? From a survey of molluskan species, Sakharov (1974) viewed transmitter specificity as one of the most evolutionarily conserved characteristics of neurons (Moroz, 2009). How might this apply to the circuits that feed T4 and T5? The evidence is obviously incomplete. T4 and T5 themselves express both *cha* transcripts and ChAT immunoreactivity, but not vesicular glutamate transporter (vGlut); both thus have a cholinergic phenotype. This might be held to support their common ancestral origin, but the weakness in this argument is that most elements of the L2 pathway are also cholinergic as well. L2 and Tm2 (Takemura et al., 2011) also express *cha* transcripts and ChAT immunoreactivity, and Tm1 is likewise ChAT positive (Shinomiya et al., 2014). In addition, Tm9 expresses both *cha* transcripts and ChAT immunoreactivity (Shinomiya et al., 2014). Neurotransmitter transcripts have been reported for neither Tm3 nor Tm4, nor for Mi1, and while L1 itself expresses glutamate-specific transcripts other cells of the L1 pathway remain something of a neurotransmitter mystery. Mi1 may be cholinergic (Hasegawa et al., 2011) and if the transmitter for Tm3, also on the L1 pathway, were likewise cholinergic, the transmitter for L1 would stand out as differing from that of L2. In that case duplication of the ME cells would seem to have occurred after the primary dichotomy between L1 and L2, and could have arisen when these cells became split in their neurotransmitter expression.

### Concluding Remarks

The existence of dichotomously antagonistic ON- and OFF- pathways has of course long been known in the vertebrate RE. There, an ON-OFF split in the visual pathways already occurs at the first synapse (Schiller, 1992), just as it also does in the fly’s LA. In both types of visual system the ON-OFF split may have evolved for rapid and metabolically efficient signaling of opposing changes in light intensity, given that light increments and decrements are each defined relative to the other, and so are assigned equal prominence in natural scenes (Schiller, 1992). Only in flies, however, does dichotomy in signaling pathways have an origin in two cell types, T4 and T5, that we propose are evolutionary siblings. Emphasizing the wider context of our proposal for ON- and OFF-edge pathways in visual systems therefore gives greater weight to the solution adopted by flies such as *Drosophila*. It remains to be determined whether during the course of evolution the dichotomy arose first with L1 and L2 in the LA or, deeper in the optic lobe, with the origin of T4 and T5 as evolutionary siblings. Looking yet further afield, it seems plausible that an evolutionary driving force toward ON- and OFF-coding pathways arose more generally, because this dichotomy appears very widely in sensory systems of many stripes. Other benefits may result from an ON/OFF split in sensory pathways, in particular that such a split may simplify downstream computations (Gjorgjieva et al., 2014).

For all that we hope it to be closely reasoned, most of what we propose is entirely speculative and unlikely to receive detailed support from comparative anatomy or the fossil record. On the other hand, we can anticipate that future comparative transcriptome analyses among identified neurons may bear with greater force on our proposals, and help address the question of how neural circuits might have arisen from among identified neurons of the same or neighboring neuropils. In parallel, we may also anticipate that more detailed studies on the development of the neurons of the fly’s ON- and OFF-edge motion pathways may also help contribute additional support for our hypothesis. For example, knowing the lineage relationships between T4 and T5 may help to consolidate further the relationship we propose for these cells as evolutionary siblings, as well as it would provide support for the relationships between neurons of their input pathways. In particular, such data could help demonstrate an evolutionary relationship between T4 and T5’s upstream partner neurons. For example the lineage and genetic relationships that we may propose from morphological evidence alone, between Tm cells such as Tm3 and Tm4, or between Mi and Tm cells, such as Mi1 and Tm1/Tm2, could help to arbitrate evolutionary relationships as robustly as we hope to have demonstrated these for their target neurons, T4 and T5. Unlike the latter pair, comparative anatomy and the fossil record are, we think, unlikely to further our understanding of the evolutionary relationships of these ME cells beyond the realm of suggestion.

## Conflict of Interest Statement

The authors declare that the research was conducted in the absence of any commercial or financial relationships that could be construed as a potential conflict of interest.
